# Complete Plastid Genome Sequence of the Brown Alga *Undaria pinnatifida*

**DOI:** 10.1371/journal.pone.0139366

**Published:** 2015-10-01

**Authors:** Lei Zhang, Xumin Wang, Tao Liu, Guoliang Wang, Shan Chi, Cui Liu, Haiyang Wang

**Affiliations:** 1 Laboratory of Genetics and Breeding of Marine Organism, College of Marine Life Sciences, Ocean University of China, Qingdao, People’s Republic of China; 2 CAS Key Laboratory of Genome Sciences and Information, Beijing Key Laboratory of Genome and Precision Medicine Technologies, Beijing Institute of Genomics, Chinese Academy of Sciences, Beijing, People’s Republic of China; University of Sydney, AUSTRALIA

## Abstract

In this study, we fully sequenced the circular plastid genome of a brown alga, *Undaria pinnatifida*. The genome is 130,383 base pairs (bp) in size; it contains a large single-copy (LSC, 76,598 bp) and a small single-copy region (SSC, 42,977 bp), separated by two inverted repeats (IRa and IRb: 5,404 bp). The genome contains 139 protein-coding, 28 tRNA, and 6 rRNA genes; none of these genes contains introns. Organization and gene contents of the *U*. *pinnatifida* plastid genome were similar to those of *Saccharina japonica*. There is a co-linear relationship between the plastid genome of *U*. *pinnatifida* and that of three previously sequenced large brown algal species. Phylogenetic analyses of 43 taxa based on 23 plastid protein-coding genes grouped all plastids into a red or green lineage. In the large brown algae branch, *U*. *pinnatifida* and *S*. *japonica* formed a sister clade with much closer relationship to *Ectocarpus siliculosus* than to *Fucus vesiculosus*. For the first time, the start codon ATT was identified in the plastid genome of large brown algae, in the *atpA* gene of *U*. *pinnatifida*. In addition, we found a gene-length change induced by a 3-bp repetitive DNA in *ycf35* and *ilvB* genes of the *U*. *pinnatifida* plastid genome.

## Introduction

*Undaria pinnatifida* belongs to the Alariaceae family and is one of the main cultivated marine seaweeds in China. As a eurythermic and perennial large brown alga, it is mainly distributed in Chinese coastal areas in the Liaoning, Shandong, Jiangsu, and Zhejiang Provinces. In addition to its primary nutritional value as a food source, *U*. *pinnatifida* potentially has therapeutic value [1]. Studies have shown that *U*. *pinnatifida* is rich in polysaccharides and has a wide range of medicinal properties, including anti-tumor, anti-viral, and immunoregulatory activities [2, 3]. Because of its wide distribution, high yields, and commercially valuable extracts, *U*. *pinnatifida* is of commercial interest to both food and pharmaceutical industries.

Plastids are semi-autonomous photosynthetic organelles found in land plants, algae, and some protozoa. Plastids carry genetic information, and their origin and evolution has long been an active area of research [4]. It is now well established that all plastids are descendants of a primary endosymbiotic relationship in which a cyanobacterium was engulfed by a heterotrophic eukaryote. This type of plastid is known as a primary plastid and is found mainly in red and green algae [5, 6]. Subsequently, these primary algal plastids spread across the eukaryotic tree by secondary endosymbiosis, whereby a photosynthetic eukaryote was engulfed by another eukaryote [7]. Such secondary plastids are mainly found in heterokonts, haptophytes, and cryptophytes and are called red-derived plastids [8].

Many molecular phylogenetic studies based on nuclear, mitochondrial, and plastid genes have been conducted to resolve the phylogenetic relationship among these three groups that contain secondary plastids [9–11]. However, resolution of the phylogenetic relationship has been difficult because methodologies and results varied significantly across different datasets [12]. An additional obstacle for resolving a topological tree for heterokonts, haptophytes, and cryptophytes is the lack of sampling data and limited integrity of datasets.

Complete organelle genome sequences provide valuable resources and information for the study of molecular ecology and evolution. As high-throughput sequencing technologies have advanced, whole genome sequencing projects have been set up for increasing number of species. As the copy number of organelle genomes is far higher than that of the nuclear genome, it is possible to assemble the organelle sequence from whole genome sequencing data. Additional plastid genomes from novel taxa will not only advance our understanding of the diversity and evolution of algae, but will also open up the possibility for genetic engineering of economically important species.

Here, we determined the complete sequence of the plastid genome of the large brown alga *U*. *pinnatifida* by using next-generation sequencing. This sequence represents the first fully characterized plastid genome from the genus *Undaria*. In addition, we performed a comparative analysis of plastid genomes of four brown algal species that will aid in the dissection of taxonomic relationships of Phaeophyceae and evolution of red-derived plastid genomes.

## Materials and Methods

### Algal Materials and DNA Extraction

Algal material was provided by the Culture Collection of Seaweed in the Ocean University of China in Qingdao. Gametophytes of *U*. *pinnatifida* (strain QD-41) were cultivated at 8–12°C in sterilized filtered seawater with nutrients (4 mg/L NaNO_3_, 0.4 mg/L KH_2_PO_4_) under fluorescent light (3000 lux; 12 h light/dark cycles). The gametophytes were concentrated on filter paper and subsequently washed three times with sterilized filtered seawater. Total DNA was extracted from drained fresh material following a modified CTAB method [13].

### Genome Sequencing and Assembly

We used approximately 5 μg of purified DNA for the construction of short-insert libraries following the manufacturer’s protocol (Illumina Inc., San Diego, CA, USA). DNA library construction and sequencing were performed at the Beijing Genomics Institute (BGI) in Shenzhen, China.

Raw sequence reads included non-plastid DNA. To determine the proportion of plastid-related reads, *S*. *japonica* (GenBank Accession No. NC_018523.1), *Ectocarpus siliculosus* (GenBank Accession No. NC_013498.1), and *Fucus vesiculosus* (GenBank Accession No. NC_016735.1) plastid genomes were used as reference sequences by using the BLAST software [14]. Subsequently, the assembly of these plastid-related reads was carried out using the SOAPdenovo software [15] with default assembly parameters. All the correctly assembled contigs were aligned and ordered to the reference plastid genomes by using the MEGA 6.0 software [16]. Gaps between the contigs and the four junction regions between both inverted repeats (IRs) and the small/large single copy regions (SSC/LSC) in the plastid genome were filled, and confirmed by PCR and Sanger sequencing by using the primers listed in S1 Table. For gap-filling PCRs, all PCR products were cloned into pMD-19T vectors with sequencing conducted for 3 clones of each product. The PCR reaction conditions included an initial denaturation step at 94°C for 3 min followed by 30 cycles of denaturing at 94°C for 30 s, annealing at a temperature specific for each primer for 45 s, and extension at 72°C for 1 min. A final extension step was performed at 72°C for 15 min, followed by a 4°C hold. Final sequences of circular plastid genomes were completed by manual assembly.

### Genome Annotation and Analysis

Annotation of the sequenced genomes was performed using the DOGMA tool [17]. Protein-coding genes were identified using BLAST algorithms to compare predicted ORFs to the NCBI GenBank database. Ribosomal RNA (rRNA) genes and coding regions were determined by sequence alignment with the known plastid genes of *S*. *japonica* and *Ectocarpus siliculosus*. Transfer RNA (tRNA) genes were identified using the tRNAscan-SE 1.21 software [18]. A plastid genome map of *U*. *pinnatifida* was produced using the OGDRAW software [19]. Sequence alignment and base composition were performed using the MEGA 6.0 software. Gene order and plastid genome sequences were aligned by the multiple alignment program of the Geneious software package (version R7 7.0.6) [20].

### Phylogenetic Analysis

Phylogenetic analysis of plastid genomes was conducted with a set of 23 protein-coding genes (*atpA*, *atpB*, *atpH*, *rbc*L, *petB*, *petG*, *psaA*, *psaB*, *psaC*, *psbA*, *psbB*, *psbC*, *psbD*, *psbE*, *psbF*, *psbL*, *psbN*, *rpl2*, *rpl20*, *rps11*, *rps12*, *rps14*, *rps19*) present in all plastid genomes publicly available in the GenBank database. Each gene was aligned individually, the whole concatenated alignment was generated, and sites containing gaps were left as is. Sequences were aligned using the MEGA 6.0 software [16] and edited manually. A maximum likelihood (ML) analysis for constructing phylogenetic trees was performed using PhyML 3.0 [21] under cpREV amino acid substitution matrices with four gamma-distributed rate categories. The MrBayes v3.1.2 software [22] was used to investigate evolutionary relationships among species based on 6,113 amino acids sequences from 43 taxa. Bayesian analysis was performed by two separate sequence analyses for four Markov chains (by using default heating values), which were run for 500,000 generations until the average standard deviation of split frequencies was below 0.01 [23]. In addition, trees were sampled every 100 generations with the first 25% of trees discarded as the burn-in. Remaining trees were used to build a 50% majority rule consensus tree, accompanied with posterior probability values. FigTree v1.3.1 (http://tree.bio.ed.ac.uk/) was used to display and print phylogenetic trees.

## Results

### Genome Organization and Codon Usage

**Fig 1:**
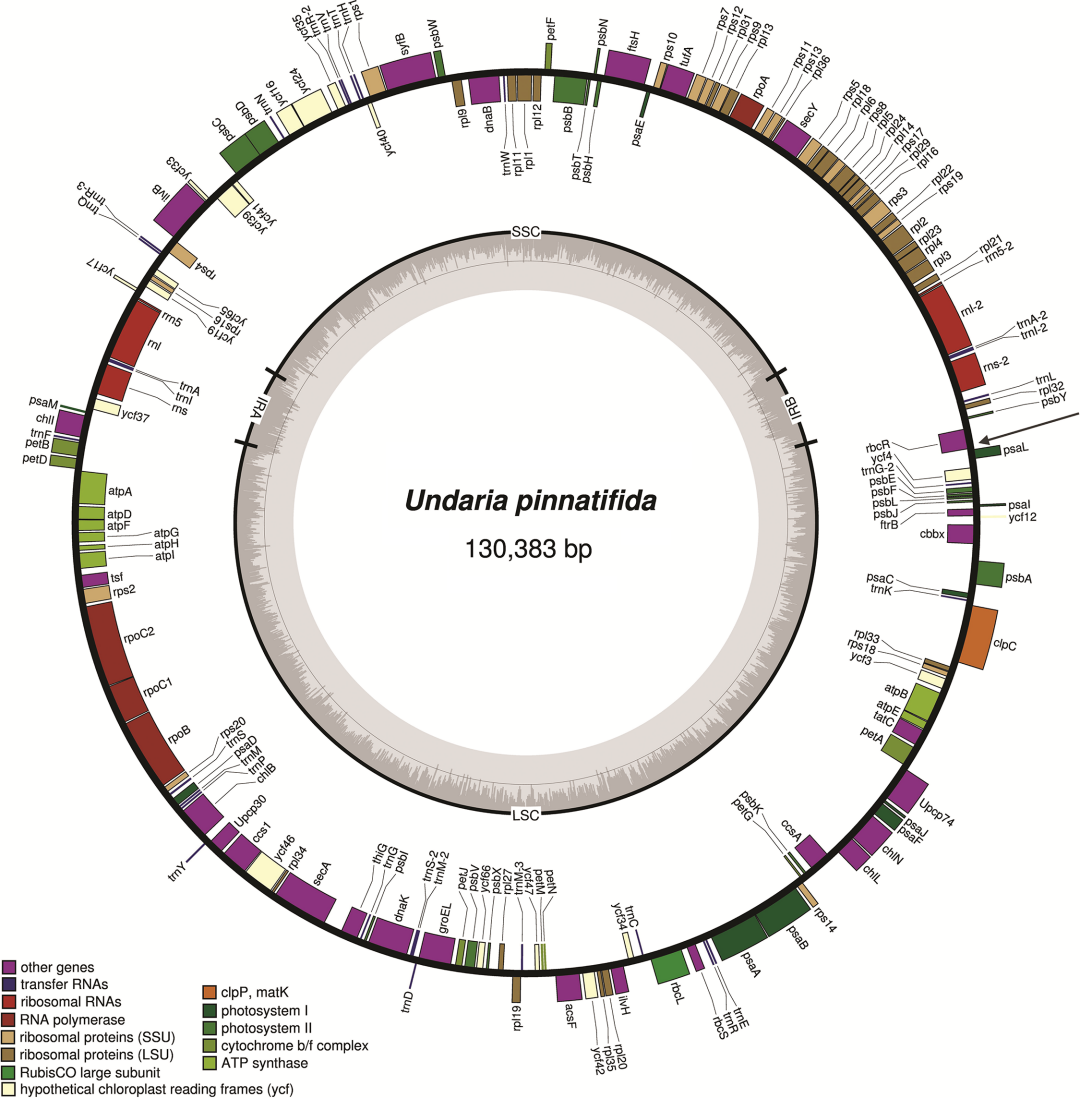
Gene map of the *Undaria pinnatifida* plastid genome. Genes on the outside of the map are transcribed counterclockwise and those on the inside of the map are transcribed clockwise. The innermost gray ring represents the GC content. The black arrow indicates the position of the stem-loop structure.

The plastid DNA of *U*. *pinnatifida* was a circular molecule of 130,383 nucleotides (nt), with an overall A+T content of 69.38%. It consisted of four typical parts: LSC, SSC, IRa, and IRb. The size of LSC and SSC was 76,598 bp and 42,977 bp, respectively, while IRa and IRb were both 5,404 bp. The *U*. *pinnatifida* plastid genome encoded 139 proteins, 28 tRNAs, and 6 rRNAs, and none of these genes contained introns. All genes were distributed in both positive and negative strands, but there was no obvious rule regarding the direction of transcription (Fig 1). Four gene overlaps were identified: *rpl23* overlapped with *rpl4* by 8 nt, *ftrB* overlapped with *ycf12* by 6 nt, *ycf16* overlapped with *ycf24* by 4 nt, and *psbC* overlapped with *psbD* by 53 nt. These overlaps are virtually identical to those found in the plastid genome of *S*. *japonica*. In an intergenic region at the demarcation point of two opposite transcriptional units, a long and stable stem-loop structure (84 bp) was identified in the plastid genome of *U*. *pinnatifida*. The secondary structure was a complete inverted repeat sequence between the *psaL* and *rbcR* genes (Fig 1).

ATG was used as the start codon for nearly all plastid genes in *U*. *pinnatifida*, except for *rps8* and *psbF*, which used GTG as the start codon, and *atpA*, which used ATT as the start codon. Comparing this to plastid genomes of three other known large brown algae, it is clear that all four genomes use ATG as the start codon for nearly all genes, and only a few genes use GTG as the start codon, mainly *rpl3*, *psbF*, *rps8*, and *rbcR*. ATT is used as the start codon of ORF76 in *F*. *vesiculosus* and, according to our results, of the *atpA* gene in *U*. *pinnatifida*. Thus, we have identified an ATT start codon for the first time in a functional protein-coding gene in a plastid genome of Phaeophyceae. The termination codons of *U*. *pinnatifida* plastid genes include three types: TAA, TAG, and TGA. Of these, TAA is the most commonly used termination codon, while TAG is present in 14–24 genes among all four Phaeophyceae plastid genomes. As shown in Table 1, the frequency of TAG as the termination codon in *S*. *japonica* and *U*. *pinnatifida*, both from the order Laminariales, was higher than that of *F*. *vesiculosus* and *Ectocarpus siliculosus*, from orders Fucales and Ectocarpales, respectively. However, the use of the termination codon TGA varied greatly among species. *F*. *vesiculosus* and *Ectocarpus siliculosus* both had more than five genes with a TGA termination codon, while *S*. *japonica* and *U*. *pinnatifida* had only one and two such genes, respectively.

**Table 1:** Comparison of codon usage of four large brown algal plastid genomes.

	Start codon			Termination codon		
Species	ATG	GTG	ATT	TAA	TAG	TGA
*F*. *vesiculosus*	136	2 (*rpl3*, *psbF*)	1 (ORF76)	113	17	9
*E*. *siliculosus*	145	3 (*rpl3*, *rps8*, *rbcR*)	0	129	14	5
*S*. *japonica*	137	2 (*rps8*, *psbF*)	0	114	24	1
*U*. *pinnatifida*	136	2 (*rps8*, *psbF*)	1 (*atpA*)	116	21	2

Numbers in the table represent the plastid gene codon usage in four species.

### Co-linear Analysis

The gene content and gene order of the plastid genomes of *U*. *pinnatifida* and *S*. *japonica*, both from the order Laminariales, were almost completely consistent. The only differences were the following: the IR region of *S*. *japonica* was 92 bp longer than that of *U*. *pinnatifida*; *S*. *japonica* encoded one more tRNA gene compared to *U*. *pinnatifida*; and gene lengths of *rps20*, *rpoB*, and *rpoA* in *U*. *pinnatifida* were 30 bp, 15 bp, and 12 bp, respectively, shorter than those of *S*. *japonica*, yet the *rpl33* gene was 15 bp longer in *U*. *pinnatifida* than in *S*. *japonica*. In addition, the plastid genome of *U*. *pinnatifida* contained an 84-bp fragment of a stem-loop structure between the *psaL* and *rbcR* genes.

**Fig 2:**
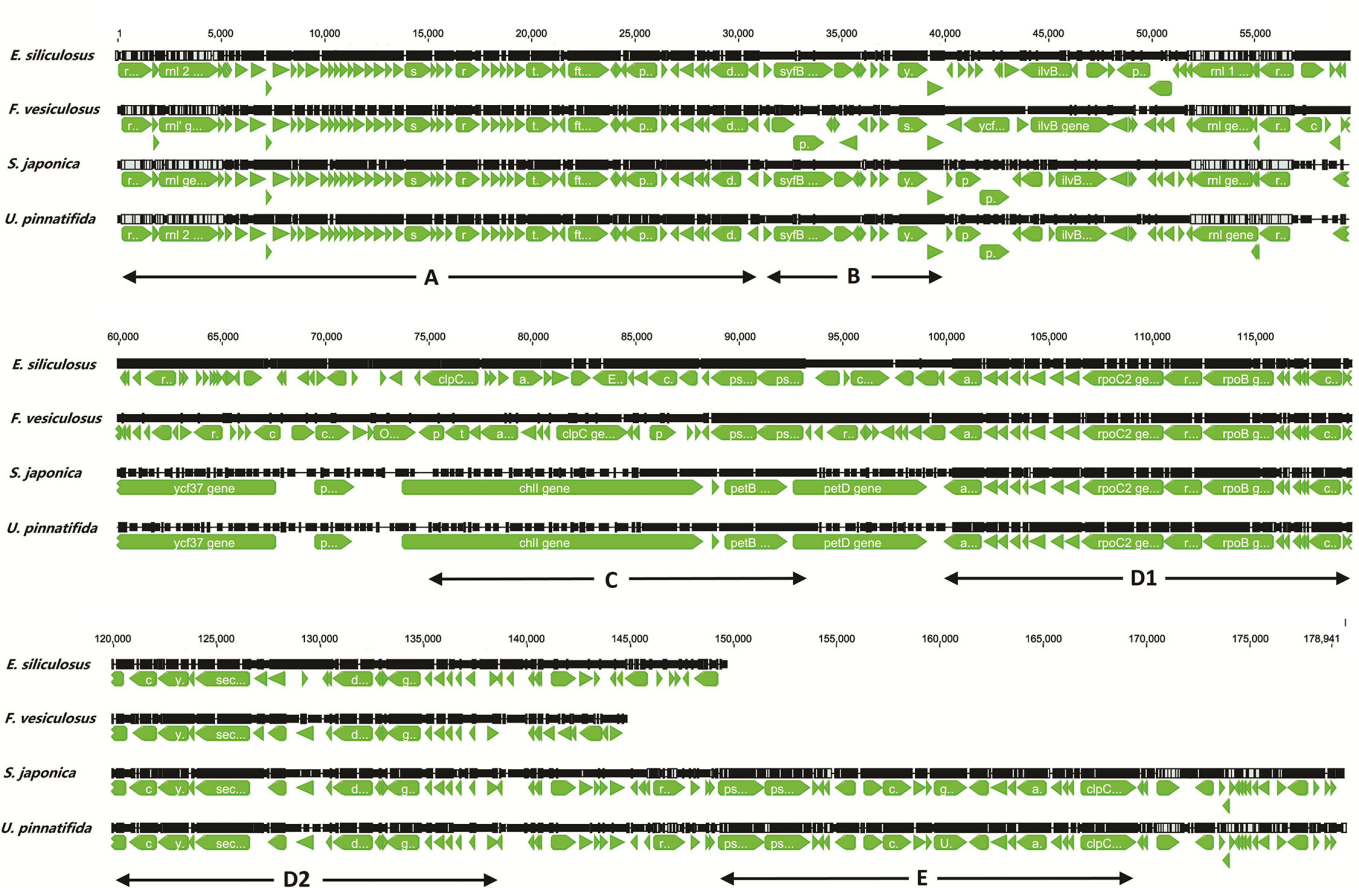
Comparison of four large brown algal plastid genomes by using the Geneious software. Letters A to E with two-header arrows correspond to the specified area of each species.

Detailed characteristics of gene order based on Geneious analyses for plastid genomes of all four large brown algae from Phaeophyceae are shown in Fig 2. The gene content and order were almost identical for *U*. *pinnatifida* and *S*. *japonica*, both of which belong to the order Laminariales, while *F*. *vesiculosus* and *Ectocarpus siliculosus* showed some differences compared with these two species. Nevertheless, there were two long and conserved gene clusters among the four plastid genomes, in which the gene order was almost exactly the same in the four species (area A and D in Fig 2). In addition, a gene cluster in area E of *U*. *pinnatifida* and *S*. *japonica* corresponded to the gene cluster located in area C of *Ectocarpus siliculosus* with the same gene content and order but in opposite direction. Furthermore, area B contained a short gene cluster identical in *U*. *pinnatifida*, *S*. *japonica*, and *E*. *siliculosus*. Overall, there was an extraordinary high linear relationship between *U*. *pinnatifida* and *S*. *japonica*, as they both had exactly the same gene content and gene order. *E*. *siliculosus* showed a higher linear relationship with these two species than with *F*. *vesiculosus*. The presence of two long gene clusters indicates a conserved evolutionary process for large brown algal plastid genes.

### Repetitive DNA in Plastid Genes

**Fig 3:**
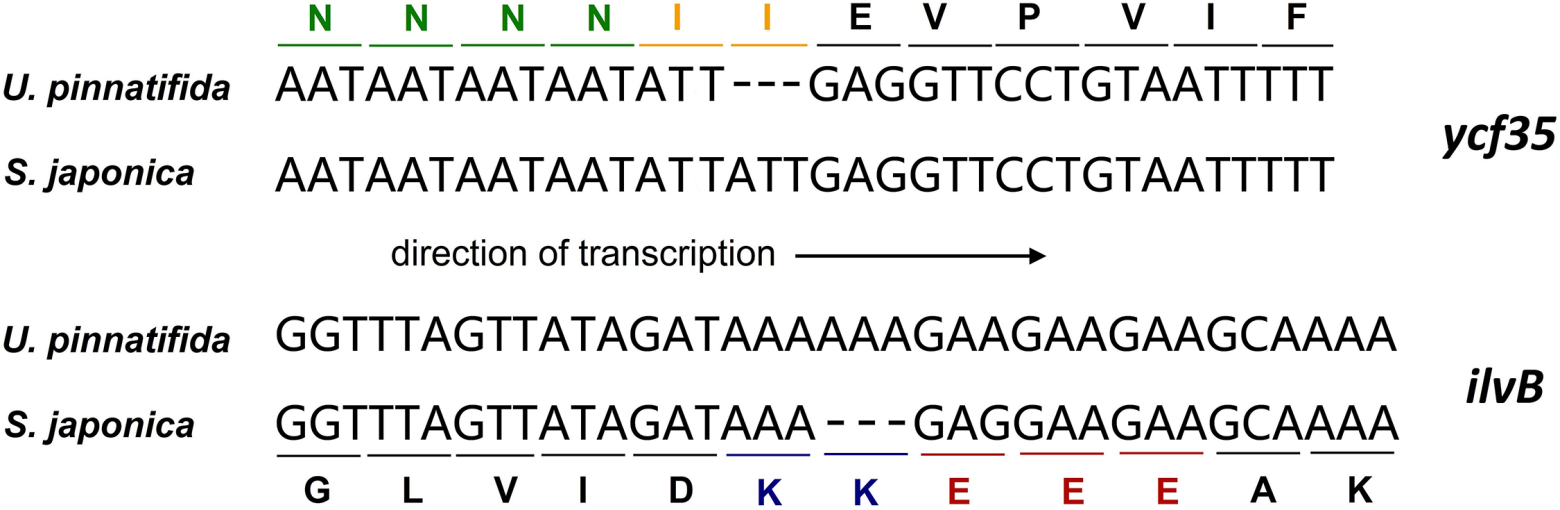
The 3-bp repetitive DNA mutations in *U*. *pinnatifida* plastid genes. Colored letters are amino acid sequences corresponding to the nucleotide sequence of each gene for both *U*. *pinnatifida* and *S*. *japonica*, and “–” represents the deletion of a base.

We found that the *ycf35* gene in the *U*. *pinnatifida* plastid genome was 3 bp shorter than that of *S*. *japonica*, because of an ATT repeat (Fig 3). However, the repetitive sequence neither affected the overall ORF structure nor caused any change to remaining rest amino acid sequences of the gene. Similar to the *ycf35* gene, another 3-bp length mutation existed in the *ilvB* gene. The *ilvB* gene of *S*. *japonica* had one less AAA repeat than that of *U*. *pinnatifida*, which again did not cause a frame-shift (Fig 3). Both mutations occurred in the region close to the tri-nucleotide repeat enrichment area. There were four AAT repeats ahead of the ATT mutation in the *ycf35* gene, and three GAA repeats followed the AAA mutation site in the *ilvB* gene. The presence of repetitive DNA may induce some genomic instability that causes minor sequence changes to occur.

### Phylogenetic Analysis

**Fig 4:**
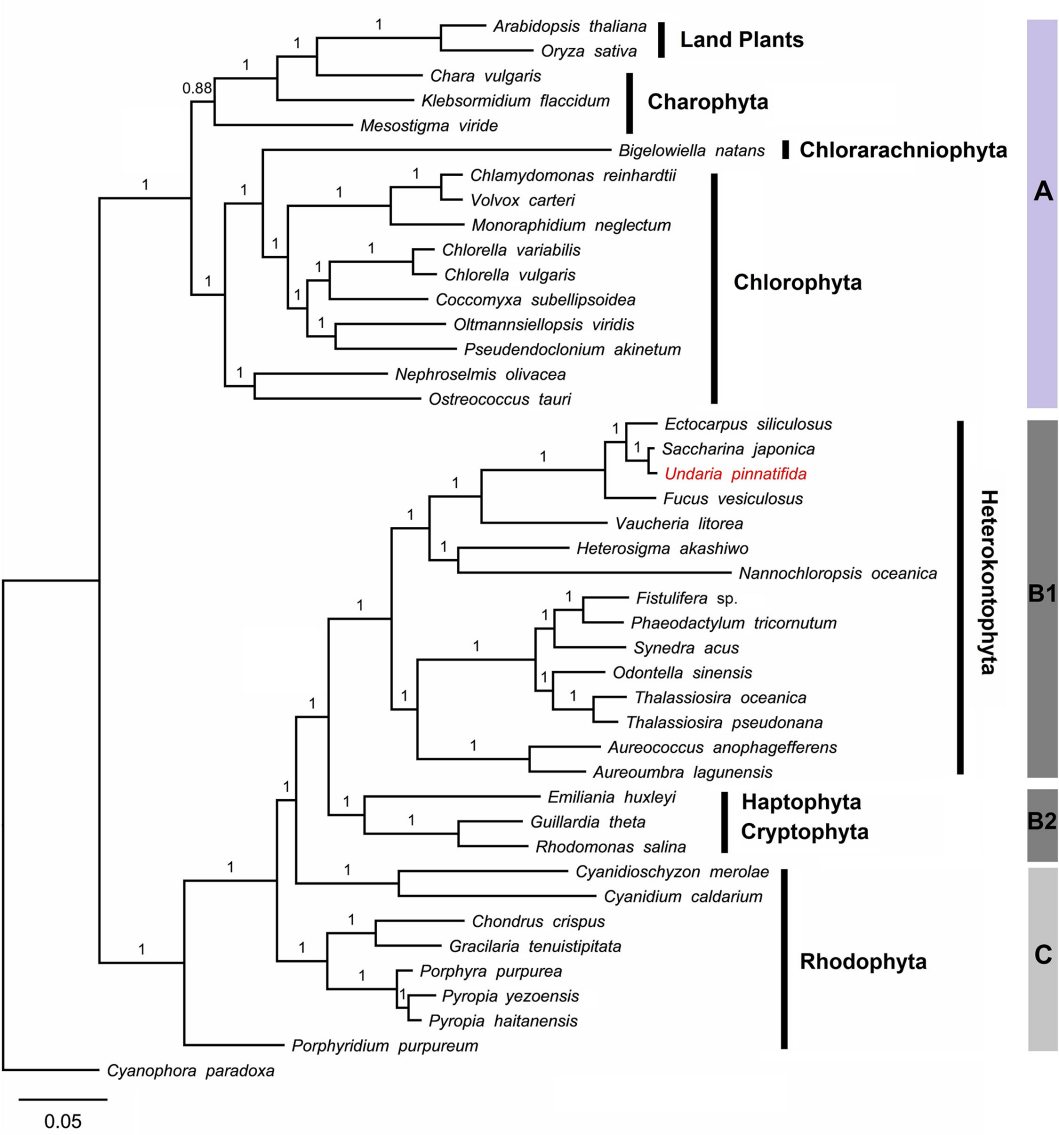
Phylogenetic tree topologies based on 23 plastid protein-coding genes inferred using Bayesian methods.

The phylogenetic dataset included 23 protein-coding genes of plastid genomes from 41 algae and 2 land plants (*Arabidopsis thaliana* and *Oryza sativa*). The phylogenetic analysis was based on the whole concatenated alignment of 6,113 amino acids. *Cyanophora paradoxa* was assigned as an outgroup. The Bayesian inference (BI) and ML analyses yielded different topologies with little difference (Fig 4 and S1 Fig). Results of both analyses suggest that secondary plastid-containing groups of Chromalveolata, including Heterokontophyta, Haptophyta and Cryptophyta, are sister groups of Rhodophyta; however, results of the ML analysis suggest that Cryptophyta and Haptophyta are separate from the rest of Chromalveolata and formed a sister group with Rhodophyta. The phylogenetic relationships based on BI analysis were consistent with results of previous studies on plastid genomes of the three brown algae [24, 25], while the relationships based on ML analysis were consistent with phylogenetic results of previous studies on the two red algal species [26]. Here, we have only presented the phylogenetic tree based on BI analysis with posterior probabilities for illustration (Fig 4). Overall, all taxa were clearly divided into two distinct lineages. Branch A includes land plants, Charophyta, Chlorarachniophyta, and the green algae Chlorophyta. Within this branch, land plants and Charophyta formed one sub-branch and *Mesostigma viride* emerged at the base of this sub-branch with a posterior probability of 0.88. The other sub-branch of branch A consisted of *Bigelowiella natans* and green algae. Branch B and C formed the other main clade. All Rhodophyta algae were grouped in branch C, with strong support for *Porphyridium purpureum* as the basal member. Moreover, there was a split between Cyanidiaceae on the one hand and Florideophyceae and Bangiophyceae on the other hand at a higher support value (100%). Branch B consisted of two clades. In clade B1, Heterokontophyta, including two taxa from Pelagophyceae and six taxa from Bacillariophyceae, formed one main group, while in the other part of the lineage, *U*. *pinnatifida* and *S*. *japonica* had a relatively high support value as the closest branch to *E*. *siliculosus*, and were further grouped with *F*. *vesiculosus*. In clade B2, *Emiliania huxleyi* from Haptophyta was grouped together with two species from Cryptophyta. These results support the conclusion that the Alariaceae family, including *Undaria*, is a sister group of the Laminariaceae family, including *Saccharina*.

## Discussion

Since the first report on the complete plastid genome of *Nicotiana tabacum* (tobacco) and *Marchantia polymorpha* (common liverwort) in 1986 [27], plastid and mitochondrial genome data have rapidly expanded in the GenBank database. Further development of sequencing technologies has greatly promoted the study of mitochondrial and plastid genomes. So far, NCBI has released more than 270 plastid genomes, including those of about 40 algal taxa. As sequencing costs decrease, whole genomes of more and more species are being sequenced. Organelle genomes are small and have a relatively simple structure. In addition, their copy number is far higher than that of the nuclear genome. Therefore, it usually is straightforward to obtain the full-length plastid genome from whole genome sequencing data, after necessary corrections and PCR confirmations. Compared to conventional homologous PCR amplification and organelle DNA separation methods for sequencing, next-generation sequencing has greatly improved both the efficiency and accuracy of organelle genome sequencing.

In this study, we identified gene length change by insertion or deletion of a 3-bp repeated sequence in two *U*. *pinnatifida* plastid genes compared to the corresponding plastid genes of *S*. *japonica*. The reading frame of both genes was unaffected, and the repetitive sequence mutations tended to occur in sequence repeat-enriched regions. It is speculated that the presence of repetitive DNA causes some instability in the plastid genome. However, we did not find variations in such repeated sequences at similar sites in the *E*. *siliculosus* plastid genome, which suggests that the 3-bp sequence mutation occurred after the differentiation of the Laminariales and Ectocarpales orders.

Comparing plastid genomes of four large brown algal species including *U*. *pinnatifida* reported in this study and three previously sequenced species [24, 25], we found a certain sequence synteny with regard to two long conserved gene clusters. However, we noticed that the gene *petL* was missing in *S*. *japonica* and *U*. *pinnatifida*, while it was present in the plastid genome of *E*. *siliculosus* and *F*. *vesiculosus*. It is well known that plastid sequences can directionally transfer to the mitochondrial or nuclear genome [28, 29], but we were unable to locate a sequence with any homology to the *petL* gene in the nuclear or mitochondrial genome dataset of *U*. *pinnatifida* (unpublished). We examined start codon usage and identified ATT as the start codon of the *atpA* gene in *U*. *pinnatifida*. ATT has not been reported as a start codon for protein-coding genes of large brown algal plastid genomes. It has been reported that ATT has lower initiation efficiency than ATG [30]. Another more commonly used start codon is GTG, which is mainly present in bacteria and in quite a few plastid genes in *Pyropia* [26]. In large brown algae, the start codon GTG was mainly used in four genes, showing a conserved evolutionary process of these plastid genes. *E*. *siliculosus* and *F*. *vesiculosus* were much more likely to use TGA as a termination codon compared to *S*. *japonica* and *U*. *pinnatifida*, which demonstrates obvious differences among large brown algal plastid genomes in the use of termination codons.

In all our analyses, plastid genomes were divided into two large groups, which show consistency with the evolution of primary plastids of red (branch B+C) and green lineage (branch A) (See Fig 4). In one clade of the green lineage, *Mesostigma viride* emerged at the base of a sub-branch containing Charophyta and land plants, which is consistent with previous research [31]. For the other clade of the green lineage, unicellular marine algae *B*. *natans* from Chlorarachniophyta was clustered within the green algae group because its plastid and nucleomorph evolved from green algae by secondary endosymbiosis [32]. Overall, the phylogenetic results indicate a clear evolutionary route from green algae to charophytes and then to land plants. The red lineage consisted of two parts, as per results of BI analysis, red-algal derived plastids including Heterokontophyta, Haptophyta and Cryptophyta (branch B) and Rhodophyta (branch C). However, as per the ML analysis, Haptophyta and Cryptophyta were separated from Heterokontophyta and formed a sister group with Rhodophyta. Within the red-algal plastids, unicellular *Porphyridium purpureum* was designated as the basal lineage ahead of the hot spring red algae *Cyanidioschyzon merolae*, in contrast to previous reports [33]. In the class Phaeophyceae of Heterokontophyta, *U*. *pinnatifida* and *S*. *japonica* show a closer relationship with *E*. *siliculosus* than with *F*. *vesiculosus*, which is consistent with our gene co-linear analysis (Fig 2) and with former reports based on mitochondrial data [34]. In summary, the phylogenetic relationships presented here confirm the existence of a red and green plastid lineage and our results based on BI analysis support the hypothesis that the red-derived plastids of Heterokontophyta, Haptophyta, and Cryptophyta have a monophyletic origin caused by a single secondary endosymbiotic event. The phylogenetic relationships resolved in this study involved plastid genomes of as many taxa as possible and these results will help us gain a better understanding of the origin and evolution of plastid genomes.

As reported previously, the mitochondrial genomes of some algae have a displacement-loop (D-loop) structure containing polymers of A and T. This D-loop is presumed to be the origin of replication, relevant for transcription [35, 36]. Compared to the mitochondrial genome, which is encoded in two main transcriptional units, the plastid genome encodes many more genes and contains several transcriptional units, as not all genes are in accordance with the major transcription direction, and two inverted repeat regions. At present, few studies have focused on the replication and transcription of plastid genomes. We report the identification of a similar hairpin structure at the demarcation point of two opposite transcriptional units of the *U*. *pinnatifida* plastid genome. This 84-bp structure was present between the *psaL* and *rbcR* genes. However, no similar sequence was found in any other known plastid genomes of large brown algae. Further studies on the transcription and replication of plastid genomes are warranted to unravel the role of this 84-bp hairpin structure in *U*. *pinnatifida*.

## Supporting Information

S1 Table Sequences of primers designed for gap filling and assembly validation. (PDF)

S1 Fig Phylogenetic tree (ML) of 43 taxa based on 23 plastid protein-coding genes. (TIF)
