# Supplementary material for: Complete Plastid Genome Sequence of the Brown Alga Undaria pinnatifida
Source: PLoS One. 2015 Oct 1;10(10):e0139366. doi: 10.1371/journal.pone.0139366 (PMC4591262; doi:10.1371/journal.pone.0139366)
Supplement: S1 Table — (PDF) [file pone.0139366.s001.pdf]

S1 Table: Sequences of primers designed for gap filling and assembly validation.

Primer Name	Primer Sequence	
	Forward primer (5'-3')	Reverse primer (5'-3')
Q-1	ATTTGGGTTTGTCATAAG	TTACTAGGTCCTGAGTTT
Q-2	TGCTATTGAAACCAAAAT	GAGTCGGAGAAAGAAGAT
Q-3	GTTACCTGACGGATGACC	TAAACTTAGCCGAGAAAG
Q-4	TGTCCGTCTTTTGTTTCT	CACGATTATGTCTTTTAGC
Q-5	ACTCTTCGGGTAATCCTT	GAAATGCCTACTGCTGGT
Q-6	GATACTCATCCCAAATAC	TAATACTGCAATAGCACA
Q-7	GTTTGGATTTCTTGATGG	TTAAAGGGATTCGTGATA
Q-8	GTCTTTGAGGCTGCTTGT	TTGGTGGCCGTTTATCTG
Q-9	GAACTATGCAAAACATCT	CTTCCTTAATAAATACCC
Q-10	TCACATTTATCCTGGGTG	GATGCCATTGTAATACTTTC
Q-11	TATCACAGAATCTACCCTTATC	GTTCAAGTCCCTCCACCC
Q-12	CTCGTAATGAAGGTCGTG	CAGCAGCATCTTGAATGT
Q-13	TGTATGAACTTGCTGAAT	AGCTTATAGAATAACGCT
Q-14	TATTTCCCTTAACTCCTG	CTATAAGTTTAGGGTTTCC
Q-15	TGTACTTAATTAGTATAGCTCAGA	TTTACCGATACCACCTTT
Q-16	TTTGAATGCGAGACAGGG	TCAGGTCCAATCGGGAAT
Q-17	GGAAGTGAAGTACCTGAG	TTTATCCATTCTGGTTTC
Q-18	GAAACTGCTTATCTATCTG	GGTTTCATAATCTTTGCT
Q-19	TTGCCTCTTGACTAGAAT	TATTTGCTCTGTGCCTAC
Q-20	CCCTACTTATGGTGCTCG	GTGGGTTGCTAACTCAATGGT
Q-21	CCTGCTTTACAACCATCC	TCACTTTCGCGTCTTTCT
Q-22	TGCTCCTTCTGTTGTTGG	TGATAGTGGTGGGCGTGT
Q-23	GGACTGCCAGTAAAAGAC	TAGTGGGCCTGATACAAT
Q-24	CTCCGTAGAATGTCATAA	CGTTTAACTTTGGTTGTT
Q-25	ACTGAGTGCGGGTTGAGA	CCAAGACGCTGCTGCTAA
Q-26	TCGAGTAGCGTTACAACA	CACGTCCTTTAGATTTCC

Q-27	TAAAATTGGCATGACGCA	CCTATTGACCCTGGAGCT
Q-28	ACCATTACCGCAGGATGT	TGGACCGAGCCCTTCTAT
Q-29	ATCCCTTTGAGGAACATG	ACTTTCACAACCAGCATT
Q-30	CATATCATAAACGAGGCA	AAAGTAGTCCGCTCTGTA
Q-31	TAAGTCATAAACAGGTGCTA	GTAAACCAAGACGGAAAT
Q-32	CTAGGGCTTAAACAAATT	ACGTTCCCATAAAGAGTA
Q-33	AAGGCTTGATATTGAACG	TTGGATGTATTTGACCGA
Q-34	GCCTTTACCTACCGTCAC	AACCACTTGGCTACACCC
Q-35	AGGCAGTGGGTTGTGATT	AAGCGTCAAGATTCCTATTC
Q-36	ATTAAACTACGGTTATTCTG	ATATGTATCGCTCCAAAA
Q-37	GCCAGAATAATAAAGTCC	GTTTCATCAAGAATCCCTAA
Q-38	AAAATCTATCTCGGAAAC	AGGTCAAATAACCCACTA
Q-39	ATAAACCGACAAACAGAG	AAGGGATACTTACATCAACA
Q-40	CCAGTTCCTCCAAGAATA	TCCCAGCATTGATGATAT
Q-41	GCTAAGCACCGAGTAAAT	CAGTGGCAATACCAGTAA
Q-42	ATTTATTCCCTGCTATGC	TTCTGCCTCATCAACATC
Q-43	ATACTGAACGCCAATCCG	TTCCTGCTGCAAGACAAA
Q-44	CCAAGTGGTAAGGCAGTG	AAACGGGTGGCAGTTATT
Q-45	CGAATGATGCTACTACCA	TTCAATGGAATACGCAAT
