# Supplementary figures and images for: Complete Plastid Genome Sequence of the Brown Alga Undaria pinnatifida
Source: PLoS One. 2015 Oct 1;10(10):e0139366. doi: 10.1371/journal.pone.0139366 (PMC4591262; doi:10.1371/journal.pone.0139366)

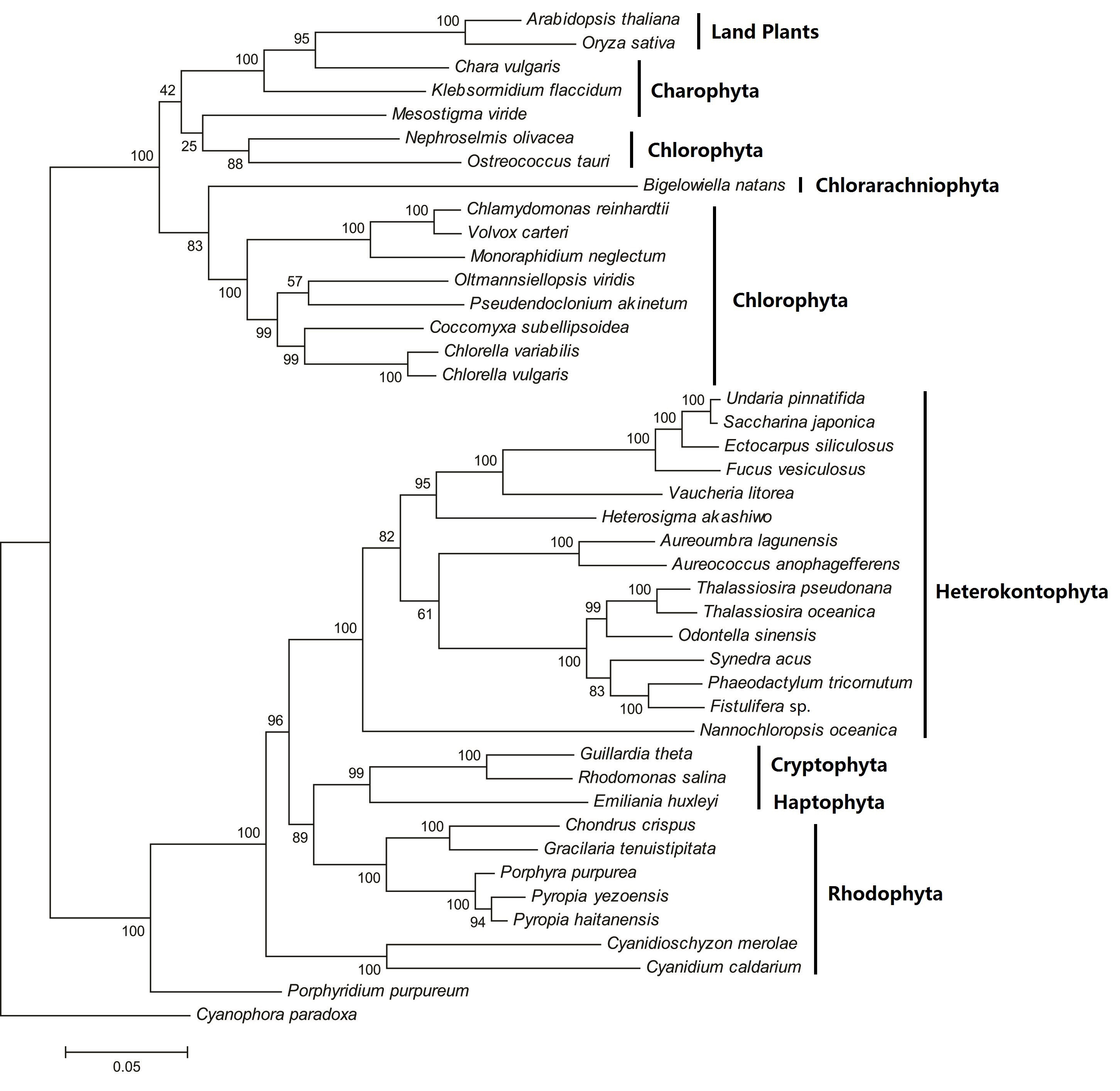

Supplement: S1 Fig — (TIF) [file pone.0139366.s002.tif]
